# Toy story or children story? Putting children and their rights at the forefront of the artificial intelligence revolution

**DOI:** 10.1007/s00146-021-01295-w

**Published:** 2021-10-06

**Authors:** E. Fosch-Villaronga, S. van der Hof, C. Lutz, A. Tamò-Larrieux

**Affiliations:** 1 eLaw Center for Law and Digital Technologies, Leiden University, Leiden, The Netherlands; 2 Nordic Centre for Internet and Society, Department of Communication and Culture, BI Norwegian Business School, Oslo, Norway; 3 FAA-Institute for Work and Employment Research, University of St. Gallen, St. Gallen, Switzerland

**Keywords:** Smart connected toys (SCTs), Internet of Toys (IoToys), Children, Artificial intelligence, Child rights, Privacy, Security

## Abstract

Policymakers need to start considering the impact smart connected toys (SCTs) have on children. Equipped with sensors, data processing capacities, and connectivity, SCTs targeting children increasingly penetrate pervasively personal environments. The network of SCTs forms the Internet of Toys (IoToys) and often increases children's engagement and playtime experience. Unfortunately, this young part of the population and, most of the time, their parents are often unaware of SCTs’ far-reaching capacities and limitations. The capabilities and constraints of SCTs create severe side effects at the technical, individual, and societal level. These side effects are often unforeseeable and unexpected. They arise from the technology's use and the interconnected nature of the IoToys, without necessarily involving malevolence from their creators. Although existing regulations and new ethical guidelines for artificial intelligence provide remedies to address some of the side effects, policymakers did not develop these redress mechanisms having children and SCTs in mind. This article provides an analysis of the arising side effects of SCTs and contrasts them with current regulatory redress mechanisms. We thereby highlight misfits and needs for further policymaking efforts.

*By accepting a new category of relationship, with entities that they recognize as "sort-of-alive", or "alive in a different, but legitimate way," today's children will redefine the scope and shape of the playing field for social relations in the future. Because they are the first generation to grow up with this new paradigm, it is essential that we observe and document their experiences*.

The Third Culture. Sherry Turkle.

## Introduction

Autonomous cars, rehabilitation robots, smart home appliances, robot toys, and virtual assistants are just a few examples of technologies that increasingly interact with humans, including children, youth, and adults, in private, professional, or public settings. Although there is an increasing interest in artificial intelligence (AI) in policymaking (Gasser and Almeida 2017; Jobin et al. 2019), how AI impacts children and their rights has received inadequate attention. According to UNICEF (2020), most major ethical guidelines and national AI strategies make superficial allusions to children and their specific needs. Moreover, country policies usually refer to children as the future AI workforce, emphasizing the need for more robust science, technology, engineering and mathematics (STEM) education to equip them with the necessary skills for an AI future. However, children increasingly use smart connected toys (SCTs), which are connected to the internet, equipped with machine learning and an ever-increasing capability to listen, observe, talk, and interact with them without appropriate guidance. An overfocus on the opportunities of these systems for children overlooks and underestimates the risks and challenges that AI systems may hold for this group (UNICEF 2020).

This article investigates the disconnect and lack of adequate redress between the side effects raised by SCTs, especially on an individual and societal level, and the regulatory means to address them. We focus on SCTs in general (Yankson et al. 2017) and the concept of the Internet of Toys (IoToys) in particular, i.e., the system enabling the interaction between SCTs. Different typologies of SCTs exist, such as the distinction of affective, functional, physical, and fictional affordances (Ihamäki and Heljakka  2018). As the word indicates, SCTs are connected to the Internet, reactive, and adaptable to changes in the environment. They can also interact with humans through voice, movement, or other modalities, with different sociality levels, i.e., they can be socially evocative and receptive and include a social interface to be more sociable (Breazeal 2003). While the focus of this article does not rest on specific technologies, the literature reviewed indicates that more and more SCTs are equipped with machine-learning capabilities to enable greater interaction (e.g., facial recognition to personalize communications with specific children).

SCTs’ target users may vary vastly, going from small children to young adults. A child is ‘every human being below the age of eighteen years unless under the law applicable to the child, majority is attained earlier [Article 1 of the UN Convention on the Rights of the Child (UNCRC)]’. Given the topic of the paper—smart toys—we however focus on younger children. Article 5, UNCRC in this respect provides that the evolving capacities of children, including their age and level of development, should be taken into account in interpreting the rights enshrined in the Convention. Unfortunately, children and their parents are often unaware of SCTs’ far-reaching capacities and limitations (Moini 2016; Albuquerque et al. 2020). SCTs’ capabilities and constraints can lead to serious side effects at the technical, individual, and societal levels such as the potential exacerbation of inherent biases for gender stereotyping, dependency, or the creation of new forms of play that blur the lines between the tangible and intangible reality (Berriman and Mascheroni 2019; Keymolen and Van der Hof 2019). These side effects are often unforeseeable and do not necessarily involve malevolence from their creators. Anticipating some of these implications may be challenging for developers.

In this article, we explore the side effects of SCTs for children and show that the redress mechanisms found in regulations and ethical statutes do not primarily have children in mind when drafted. Indeed, although the literature is rich in initiatives promoting reflection upon the consequences and outcomes of technological research and development (R&D) and fostering the incorporation of such considerations into the research or the design process, a child-specific focus seems to be lacking (Eden et al. 2013; Stahl et al. 2014). Hence, we notice a discrepancy between the adequacy of redress mechanisms provided in such standards, regulations, and initiatives concerning the target audience of SCTs.

We follow a critical socio-legal approach that combines conceptual analyses of the side effects of SCTs with an in-depth interpretation of existing legal frameworks and their shortcomings. Particularly, we propose that policymakers and legal scholars should take a children’s rights approach to regulating SCTs. This socio-legal children’s rights approach acknowledges the agency of things and concepts and the complexity of interactions within an ecosystem (Nash et al. 2019) with the goal to describe these relationships among human actants (in our case children) and artificial ones. It allows us to take stock of the arising side effects and elaborate on the legal remedies that have emerged, highlighting thereby the misfits and needs for further policymaking efforts.

Aside from an introduction and conclusion, this article contains three main sections. After introducing the concept of the IoToys, using concrete examples and explaining SCTs' characteristics in Sect. 2, we discuss the side effects of such technologies in Sect. 3. Side effects are unintended consequences that arise from the technology's use and the interconnected nature of the IoToys. These effects may lead to unforeseen, underestimated, or overlooked harms. Part of the literature has focused on the challenges of SCT, referring mostly to security (Shasha et al. 2019). However, aside from side effects arising from a technical level, we explore how SCTs’s design make children more prone to like and trust them, allowing companies and third parties to exploit such vulnerabilities. Side effects arising on an individual level include obsessive use and dependency of children on their SCT and children over trusting their devices to have their best interest in mind even when doing so is not advisable. On a societal level, these individual side effects accumulate, potentially leading to normalizing surveillance via SCT, enabling the creation of even more accurate profiles of children and predictions about their future behaviors (Keymolen and Van der Hof 2019; Yankson et al. 2017). Moreover, as with all digital technologies, a digital divide of children who can afford digital companions and ones who cannot is becoming increasingly visible (Mascheroni and Holloway 2019). After mapping the side effects of the growing interconnectivity of toys for children, we bring regulatory provisions that could mitigate the described effects of SCTs (Sect. 4). We elaborate on the children’s right approach, privacy, security, transparency and fairness provisions within data protection law, the commercialization of play, diversity and societal implications. We close the article by stressing the urgent need for policymakers to put children and their rights at the forefront of the AI revolution.

## Internet of Toys: the rise of smart connected toys

Toys are becoming increasingly media-like and computer-like, where physical and digital aspects come together (Berriman and Mascheroni 2019). The first SCTs were the Tamagotchi (a digital pet), which appeared in 1996, and Furby, a robotic toy with fur (in 1997). Since then, SCTs have become much smarter, with Pleo a toy dinosaur and Barbie Talk as examples that show how SCTs enable new forms of play, so-called connective play (Marsh 2017), and have novel affordances (Goldstein 2013). However, SCTs also raise specific challenges, some of which we will discuss below. Peter and colleagues (2019) offer an overview of the defining elements of smart toys, differentiating them from connected toys. The latter connect to the Internet, but are not necessarily smart. By contrast, smart toys do not necessarily have to be connected to the Internet, but are considered ‘smart’ because they can interact with children through voice, movement, or haptic adaptability. If smart toys connect to the Internet, they form a network of toys, which we refer to as the IoToys, a subset of the Internet of Things (Peter et al. 2019). While we acknowledge that there is no agreed definition for SCT (Albuquerque et al. 2020), for this paper, we use the definition of Hung et al. (2016, p. 71), who describe SCTs as cyber-physical devices "consisting of a physical toy component that connects to a computing system with online services through networking and sensory technologies to enhance the functionality of a traditional toy." Thus, we are interested in smart toys connected to the Internet (rather than non-smart toys connected to the Internet or smart toys not connected to the Internet).

In more detail, SCTs have six defining attributes, which are more or less pronounced depending on the specific toy in question (Peter et al. 2019): they (1) are powered by energy (e.g., a battery), (2) rely on sensors, (3) are software-controlled, (4) are interactive (i.e., they can react to inputs and are not static), (5) possess mobility, (6) and are embodied, rather than virtual. Given these characteristics, SCTs come with dedicated affordances such as liveliness, portability, and affective stickiness (Berriman and Mascheroni 2019). For SCTs, anthropomorphic, zoomorphic, caricatured, and functional embodiments (Fong et al. 2003) play a crucial role across applications. SCTs should be understood threefold: first, broadly as physical devices and artifacts that help children communicate; second, as the surrounding practices and activities emerging from interactions with SCT, including the use and practice-based aspects; finally, as the ecosystem surrounding and enabling SCTs, such as organizations, the institutional and social environment of the application, including the home (Mascheroni and Halloway 2019 referring to Lievrouw and Livingstone 2006). Overall, compared to traditional toys, SCTs function as black boxes with monitoring and data practices mostly inconspicuous to parents and children (Nash et al. 2019). Because of their networked character, they present a form of hybrid ownership, leaving them under the toy company's control even after having been purchased (Keymolen and Van der Hof 2019). More specifically, given their potential for dataveillance and marketing to children, they provide an unprecedented amplified commercialization of play, including the commodification of children's identities (Van der Hof et al. 2020).

Given their characteristics, SCTs resemble social robots, so that many ethical, legal, and social challenges arising from social robots also apply to them (Fosch-Villaronga et al. 2020a; b). However, compared to social robots, SCTs tend to be less horizontally and vertically integrated (Peter et al. 2019). In other words, a smart toy will typically have a smaller number of characteristics (out of the six ones mentioned) present compared with a social robot (horizontal integration), and each of the features might not be as strongly developed (vertical integration). In social and legal terms, SCTs are not social robots, which can be used either by children and adults. SCTs are developed and used primarily by children, a group that requires specific protection and consideration. In interviews with parents of children owning a SCT (Hello Barbie, CogniToys Dino), McReynolds and colleagues (2017) also discovered that connections were made between SCTs and other Internet of Things devices like smart speakers.

## Side effects of the Internet of Toys

In general, side effects are broad, secondary, unplanned and mostly undesirable issues and concerns that arise from a phenomenon. Side effects often have an adverse impact on an individual, group of individuals, or society at large (Tamò-Larrieux 2021). While side effects are typically undesirable, instances of more positive effects can be noticed. For example, children diagnosed with autism spectrum disorder react quite positively to embodied technologies such as robots (Cabibihan et al. 2013) and SCTs (Laurie et al. 2021), providing promising avenues for their learning and wellbeing more generally. In the context of SCTs, side effects result from the use by children. Precisely because SCTs target children, a special focus on the impact of such devices is needed. Doing so requires a thorough analysis of the side effects of SCTs (Sect. 3), before being able to contrast the arising issues with existing regulatory frameworks (Sect. 4; sub-Sect. 4.1). This then allows us to see shortcomings of current legislation and potential ways to address them (Sect. 4; sub-Sect. 4.2).

SCTs' side effects can be broadly differentiated into three interconnected clusters. The division into clusters is adapted from Liu (2018), who proposes to analyze three levels of power structure with respect to AI: power exercised over individuals and groups, power impacting societal development, and power involving existential threats to humanity. In his analysis, Liu (2018), relying on Stephen Lukes' analysis of power, treats AI as "problems of power" (p. 199). While such a focus on power already subsumes a normative outlook, we follow a more descriptive, socio-legal approach in this article. Therefore, we adopt a primarily descriptive approach that aims at mapping the arising side effects of SCTs. This approach aligns with socio-technical studies and actor-network theory (ANT), which use descriptive, constructivist approaches that acknowledge the agency of objects and concepts and describe the relationship among social and technical actors (Latour 1987). Such an approach is well suited to map the SCT and IoToys ecosystem and describe the resulting side effects. Previous research has employed ANT to complex technological systems such as e-health (Muhammad and Wickramasinghe 2014), the introduction of telepresence robots (Beane and Orlikowski 2015), the privacy ecosystem of healthcare robots (Lutz and Tamò 2018), and trust implications of smart toys (Keymolen and Van der Hof 2019). The goal of describing the relationship on various levels among technology and individuals is not to determine precise, cause-and-effect relationships, but much more to illustrate the link and relationships among human and material actants within a network (an actant being anything that can influence an event) (Krieger and Belliger 2014; Latour 2005). Such a broad, descriptive approach is useful at this stage as it allows taking an ecosystem perspective in a field where existing research has narrowly focused on harms caused by SCTs (e.g., security breaches) (Shasha et al. 2019).

Building upon this theoretical framework and aligning our work with similar research (e.g., Nash et al. 2019), we analyze the side effects within a socio-legal approach that centers on the challenges and needs for children. Inasfar, our approach can be seen as corollary to current socio-legal approaches with a more narrow focus. We believe that taking such a more narrow approach helps contextualize and raise issues that are specific for the child–SCT interaction that might be missed by taking a broader lens.

**Fig. 1:**
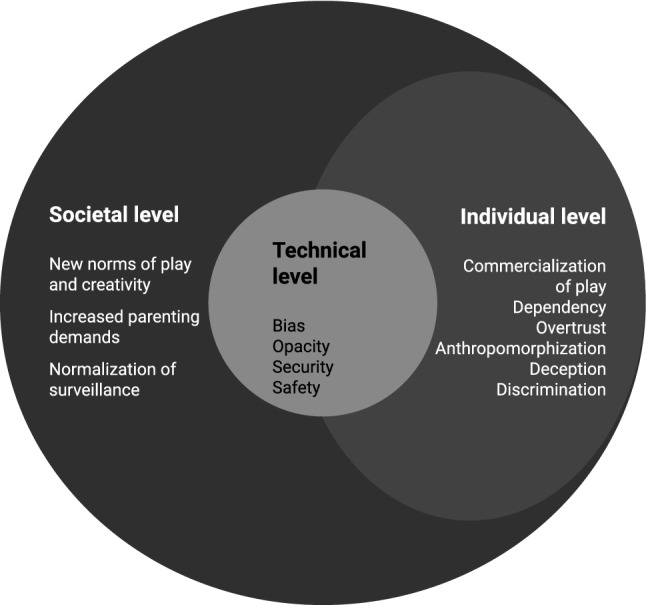
Smart connected toys and their interrelated side effects at the technical, individual, and societal levels

Two of our clusters of side effects roughly correspond with Liu’s (2018) levels of power structure. We analyze side effects on an individual level and on an aggregate, societal level that aligns with Liu’s (2018) focus on societal development. However, in contrast to Liu (2018), we forgo the analysis of existential threats as this would not match the case of SCTs. However, we introduce a new cluster, namely the side effects on a technical level. These technical side effects are significant because SCTs are more concrete technology than AI in general, as analyzed by Liu (2018). Figure 1 shows these three interconnected levels and the identified side effects that will be discussed in more depth.

All these side effects affect one another and can have repercussions in the other levels. The societal level, for instance, is heavily intertwined with the technical level and the individual level, and considerable work has tried to conceptualize the interplay between the micro-level and the macro-level (e.g., Coleman 1990). However, detailed discussion on this would go beyond the scope of the paper and we mainly use the distinction as a structuring device. First, since SCTs are man-made constructed devices that sense, make decisions, and act within their environment (e.g., at home or school), similar to social robots (Lutz and Tamò 2018), side effects can arise at the technical level. These side effects can range from security to safety issues, i.e., whether the SCT is safe to use; but also to other more profound issues, such as the replication and exacerbation of inherent biases such as gender stereotypes, which is mostly underexplored in the context of SCTs (Sathyamurthy 2018). Second, SCTs have side effects on an individual level, when children interact with SCTs directly. Since the interaction can be physical or social, it can entail side effects that navigate from dependency to trust and deception. Moreover, since SCTs underpin the commercialization of play, such an interaction is not reserved for the SCTs and the child only. It also relates to the larger business ecosystem in which a SCT has been created. Here, the SCT may not be the only relevant unit but cloud services, third parties, and other relevant actors (e.g., the school that sets up specific educational exercises) might matter as well (Fosch-Villaronga and Millard 2019). Third, the continuous use of interconnected toys may imply a societal change, in terms of changing norms and values (e.g.., what does it mean to play?), the emergence of new forms of play (what are the effects of augmented commercialization of play on children?), and larger behavioral trends (i.e., parental attention time, mediated-sociability, alienation, dataveillance).

### Technical level

SCTs store, process, and transmit data just like computers. They have onboard sensors similar to smartphones, other smart devices, some wearables, and IoT devices. SCTs can also interact with users in different ways, including via text or voice, as much as smart personal assistants, such as Cortana, Siri, and Alexa (Rueben et al. 2017; Fosch-Villaronga 2019). One example is Hello Dreamhouse, from Barbie, which is an interactive doll house.^1^ Another example is the Fisher-Price Smart Toy Bear, which is ‘an interactive learning friend with all the brains of a computer, without the screen.’^2^

SCTs easily store and collect images of their users' physical activity status (walking, running, sitting), location history information through a camera, microphone, GPS, and several others (Pontes et al. 2019). One of the side effects of these technical functionalities is the existence of penetration points allowing for larger attacks, which are often underestimated due to SCT's innocuous presentation but that users are unprepared to mitigate if they occur (Jones and Meurer 2016; Streiff et al. 2019). Streiff et al. (2019) showed how easy it was to obtain root access to the smart bear toy from Fisher-Price. The researchers captured live pictures and videos and installed remote access software, allowing private recordings over WiFi network connections without user knowledge or permission. Although the literature acknowledges the potential adverse effect of these technical functionalities and some reviews have been conducted (Pontes et al. 2019), this research field is still largely compartmentalized, lacking a comprehensive systematic study covering SCT vulnerabilities at the technical level (Albuquerque et al. 2020). In particular, what remains largely understudied is how these vulnerabilities affect children (Streiff et al. 2019).

One of the few large studies on the topic of security vulnerabilities was conducted by Shasha and colleagues (2019), who concluded that the three most frequently found risks refer to (1) how the data are used, retained, and disclosed, (2) the need for consent for any data operation to be performed, and (3) the need for broad transparency by SCT manufacturers, so that the child's guardian has a comprehensive understanding of what such an interaction between the SCT and the child entails. The authors also noted that existing studies pay insufficient attention to aspects of integrity, availability and accountability.

Importantly, the data processing of SCTs is often not transparent, in particular for the target audience of children. The side effect of opacity, as the absence of transparency, has been well documented in literature on social robots, AI and automated decision-making systems (Burrell 2016; Felzmann et al. 2019a, b, 2020; Larsson & Heintz 2020; Wachter et al 2017; Walmsley 2020; Zerilli et al. 2019). SCTs are complex cyber-physical products, whose inner workings might not be clear and transparent, neither for children, nor for parents and adults in general (Keymolen and Van der Hof 2019; Fosch-Villaronga et al. 2018a, b). This reasoning may make sense to a company that manufactures toys that support real-time social interaction and that are expected to appear as a friend to children (Jones and Meurer 2016). Opacity can lead to functional issues (e.g., a SCT stops working, and it is unclear why) and social ones (e.g., parents unable to tell children what is going on with a smart toy). Existing literature discusses the opacity of SCTs, and smart systems for children more broadly, in the context of privacy and data rather than the general functioning of the technology (Berman and Albright 2017; Holloway 2019).

In addition, SCTs might be developed based on inaccurate assumptions or biased data that favors certain ethnicities, genders and social classes (Holloway 2019). Given their adaptability and interactivity, the risk is that SCTs work better for distinct audiences than others. In particular, disabled children, such as those with a speech disorder, risk being excluded from their use (Jadi 2019). In addition, gender stereotyping has been identified as an issue with SCTs. Keymolen and Van der Hof (2019, p. 152) describe how “Hello Barbie is much more interested in talking about clothes and toys, whereas the smart robot i-Que (targeting boys)” addresses topics such as lasers and science. Thus, the design of toys for specific gender groups risks fixating children on a certain identity.

### Individual level

Side effects at the technical level may have repercussions at the individual level when the child interacts with the SCT. For instance, since SCTs support social interaction, a child may be psychologically afraid if a compromised toy suddenly has a distressing voice and displays disturbing, violent, or pornographic content (Shasha et al. 2019). If the child entrusts the toy and shares personal information, any security breach could harm the child in different ways.

SCTs support different types of interaction, including physical and social, and may evoke social responses from the children or involve psychosocial elements like trust (Di Dio et al. 2020). Supporting individual or multiplayer options, these interactions have a spatial and a temporal dimension too. Spatial because they may happen in known environments to the child (e.g., home or school), unknown (in malls, for instance), or in different locations at the same time (for multiplayer option). The temporal element refers to whether the interaction is sporadic or geared toward supporting longer-term engagement over time, but also with respect to the age of the child, which can very much play a determinant factor on the risk type and mitigation strategy. The former is often based on emotion and memory adaptations that designers manipulate to combat the decline of a child’s interest (Ahmad et al. 2017). In this respect, the literature alerts that, given our human tendency to form bonds with the entities with whom we interact and the human-like capabilities of these devices, children will have strong emotional connections when immersed in connected play (Leite et al. 2013), leaving them in a vulnerable position.

Due to their affordances and design, SCTs can become deeply integrated into children’s lives, with the potential for dependency (Brito et al. 2018) to the extent of potentially leading to social isolation (Cagiltay et al. 2014). In ethnographic research on smart toy adoption and domestication in Portugal, Brito et al. (2018) found that children engaged more with the toys than the real world, with parents observing alienation of children “from family members, not paying attention to them and these situations are sometimes the subject of family discussions” (Brito et al., p. 10). Adjacent research has shown that children develop stronger bonds with a physical robot than a virtual avatar (Sinoo et al. 2018). One reason for the ability of SCTs for such dependency is the *anthropomorphization* aspect of such gadgets (Darling 2012). The allocation of social-interactional intelligence to SCTs (typically via gaze and facial expressions) makes task capabilities more intelligible for children (Shamekhi et al. 2018) and increases engagement and gullibility. Aware of how the design of SCTs affects the engagement of children, SCTs designers and producers may exploit children's gullibility for more engagement at best (Wagner and Akin 2011; Westlund and Breazeal 2015) and manipulation at worst, e.g. by adding dark patterns and marketing strategies into the interaction with SCTs (Keymolen and Van der Hof 2019; Van der Hof et al. 2020). However, and on aggregate, digital technology use more broadly among young people tends to have little or no effect on mental health issues, dependence and aggression (Bell et al. 2015; Vuorre et al. 2021). Most children and adolescents seem resilient in that regard “and there is currently no evidence from neuroscience studies that typical internet use harms the adolescent brain” (Bell et al. 2015). Nevertheless, this does not rule out specific risks of SCTs, as Nash et al. (2019) discuss with reference to three specific types of risks: content-related risks, contact-related risks, and conduct-related risks. Their content analysis of media coverage of SCT-related incidents revealed that the major topic was privacy and that “[t]here were no reports of directharms of the three types outlined above” (p. 15).

Another side effect that emerges from both the deception and anthropomorphization is *overtrust*. Overtrust arises when children overestimate the capacities of a SCT, develop unrealistic expectations regarding its role and functionality or are insufficiently aware of the risks. The question is to understand who is in the position to elucidate whether this is a problem. Children are especially susceptible to overtrust risks because children cannot adequately assess the hazards of using sophisticated technological devices (Borenstein et al. 2018). Parents, who would usually be in the position to give such an assessment, are also often very emotionally invested in the technology as a solution for their child, such that they may not adequately identify and evaluate the risks associated with the use of a robot.

SCTs also contribute in various ways to the commercialisation of children's play. They provide a play environment in which marketing strategies can be used to make children sensitive to brands and to encourage them to buy them. In addition, SCTs can make use of dataveillance strategies to improve products based on children's data or to draw up consumer profiles of children (Nash et al. 2019). In all cases, the commercial interest of the company is the driving force rather than the child's best interest (Van der Hof et al 2020; Verdoodt 2019).

### Societal level

Compared to the individual level side effects discussed in the previous sub-section, societal level side effects occur on an aggregated level and affect whole societies or sub-groups within them (e.g., based on social categories such as gender, race, socio-economic status, and class). They also include long-term trends (e.g., value changes) and developments that trigger policy responses and affect a large number of people. The COVID-19 pandemic is an example (Newlands et al. 2020).

A first societal level side effect of increased adoption of SCTs is the spread of surveillance (Zuboff 2019), with the normalization of surveillance among a particularly vulnerable and protected group as a particular concern (Barassi 2018). Pinto and Nemorin (2014) stated that SCTs like the Elf on the Shelf introduce surveillance in playful and uncritical ways with potentially powerful, wide-ranging ramifications. While ample research has discussed the increasing pervasiveness of surveillance due to big data and Internet-based technologies more generally, introducing useful concepts such as datafication (Sadowski 2019), dataveillance (Van Dijck 2014), surveillance capitalism (Zuboff 2015, 2019) and data capitalism (West 2019), less research has reflected on the implications of digital surveillance for children. However, an emerging discourse on parenting, family and children in the digital age touches on such aspects (Barassi 2017a, b, 2018, 2020), showing how “intimate surveillance has become a popular practice among parents” (Lupton and Williamson 2017, p. 783). Accordingly the normalization of surveillance through SCTs and other AI-based applications could lead to more streamlined childhoods with less room for creativity and self-development, especially if children (and their parents) take these technologies for granted and are locked-in into their eco-systems (Gaspar et al. 2018). Within a developmental trajectory, the normalization of surveillance of children could lead to more habituation and acceptance of surveillance technologies once the children reach adult life.

At the same time, the increased introduction of SCTs could pose additional demands and burdens on the parents, many of whom might lack the literacy to “prescribe” such technologies responsibly, if this is even possible in their full functionality. Livingstone and Blum-Ross (2020a) describe how many parents in the UK are struggling with parental guidance on digital technology use and find popular advice (such as not more than two hours screen time per day) insufficient and confusing. Instead of guidance on how much time children should spend with digital technology, parents would like to have more information on which activities and use modalities are recommended and which ones should be avoided. However, such information is not easy to come by. A side effect is thus that the parents face additional responsibilities that might lead to more stress and strain their wellbeing, particularly in times of the COVID-19 pandemic (Brown et al. 2020; Livingstone and Blum-Ross 2020b).

In addition, there are intersectional side effects and implications regarding access to and use of SCTs. For those technologies that advance the children’s wellbeing, there is no requirement to access them. For instance, pediatric access to exoskeletons lags far behind that of adults (Fosch-Villaronga et al. 2020a; b). In a recent article, we query the possible reasons for this variability in access, explicitly focusing on children, who constitute a categorically vulnerable population, and also stand to benefit significantly from the use of this technology at this critical point in their physical and emotional growth. Children from disadvantaged backgrounds might have lower quality SCTs than those from more privileged backgrounds, focusing more on entertainment and pacification than education and learning. This disadvantage might become stark when smart toy based learning becomes a new norm supporting or even supplementing current learning approaches (Cagiltay et al. 2014). Such differences in capital-enhancing Internet use based on socio-economic status have been demonstrated for the general population (Zillien and Hargittai 2009) but less evidence is available for children (although the existing evidence seems to point in the same direction, see for example Zhang 2015). However, digital inequalities research has shown considerable differences in access to digital technologies, skills and use among children from different backgrounds, cautioning against digital nativism and techno-optimist stances that connected technologies will reduce inequalities among children (Livingstone and Helsper 2007; Santillana et al. 2020; Wilkin et al. 2017). SCTs pose a risk of exacerbating existing inequalities from a young age on, particularly since SCTs are mostly employed in private households and during children’s sparetime, rather than in an institutional setting where emerging divergences can be easier detected and approached. In related terms, SCTs might also reinforce gender stereotypes and challenge gender equality attempts (Francis 2010 on the genderedness of traditional toys; Keymolen and Van der Hof 2019 on SCTs). With regard to the datification of childhoods more generally, Lupton and Williamson (2017, p. 787) write: “A significant risk that children’s opportunities might be narrowed by the assumptions encoded in algorithmic processes is raised by such techniques,” a point Barassi (2020) also raised.

New norms of play and creativity could also emerge due to SCTs. While the hope is that children find more ways of self-expression, learning and personal growth, a challenge or side effect could be that typical and prescribed uses of SCTs frame the child more as a consumer rather than an active learner. Moreover, these new forms of play may also be inherently commercial, resulting in a commercialization of play and commodification of children’s identities as mentioned previously in relation to individual level side effects. However, there is also a societal side to it in the sense that playful activities are going to be seen as something of an economic benefit rather than an activity that is valuable in itself. What is more, children engaging in play are perceived as economic commodities themselves. It puts capitalist values above meaningful human interaction and personal development (see generally, Nussbaum 2016) and may thus stimulate consumerism and materialism in ways that are increasingly difficult to avoid or even detect by children or parents for that matter (Van der Hof et al. 2020) and that do not necessarily contribute to the wellbeing of children or may even be harmful to them (Fikkers et al. 2017).

## Addressing the side effects of the Internet of Toys: selected regulatory issues

### Overview of relevant regulatory frameworks

Policymakers in the European Union (EU) have become aware of the potential benefits and challenges of automated decision-making systems, and have discussed ways to foster their prospects and remedy their downsides. To this end, the EU has set in place ethical guidelines for the development of AI (HLEG AI 2019), intended to ‘foster responsible and sustainable AI innovation in Europe’ and generate trust in AI innovation. The High-Level Expert Group on AI (HLEG AI) highlights that lawful, ethical, and robust AI will ensure trustworthiness in these systems and promote ‘responsible competitiveness.’ In a white paper ‘On Artificial Intelligence—A European approach to excellence and trust,’ the European Commission (EC) (2020a) mentioned children twice, in footnotes. Other pieces focusing on AI, policy, and children make little reference to the dignity of children or the adverse effects of AI on children (UNICEF 2020).

Likewise, the private sector is exploring the market potential of automated decision-making systems and expanding on principles that should guide the development of AI services (Hilligoss and Fjeld 2019). These principles aim to ensure that AI is responsible and respects privacy, responsibility and accountability, safety and security, transparency and explainability, justice, fairness and equity, non-discrimination, non-maleficence and beneficence, human control of technology, professional responsibility, freedom and autonomy, trust, sustainability, dignity, and promotion of human values such as solidarity (Jobin et al. 2019). However, attention to children is not very much present.

Aside from such guidelines and strategies to mitigate side effects of new implementations of technologies, we can rely on established regulatory frameworks, such as the Convention on the Rights of the Child (including the general comment on children’s rights in relation to the digital environment), privacy and data protection regulation, and more recently proposals to regulate AI itself. First, the Convention on the Rights of the Child (CRC) can be taken as a standard to protect children’s rights. The CRC ensures children’s protection, participation and development rights. Although states are usually the recipients of human rights treaties, the United Nations Human Rights Council increasingly draws attention to the responsibility that corporations, sectors, and industries worldwide have for respecting human rights (OHCHR 2012), including children’s rights (UNICEF 2012). In other words, companies have the responsibility to identify, prevent, mitigate, and remediate adverse impacts on human rights (UNICEF 2014). In this respect, it is clear that companies are responsible for implementing the best interest of the child principle (article 3 UN CRC). Moreover, a General Comment by the committee on the Rights of the Child (No. 25, 2021) further stresses the importance of the best interest principle as well as the need to hear from children and respect their views when it comes to regulating matters that affect them.

How states will adopt these guidelines remains to be seen. Second, remedies against side effects such as privacy and security breaches, opacity, and potential biases in data processing can be partially found in the General Data Protection Regulation (GDPR). The GDPR contains overarching principles when personal data are being processed, such as the principles of transparency, fairness, data minimization, and data security. Combined with the data protection by design and default principles (Art. 25 GDPR), the fundamental processing principles create the obligations for SCTs developers to design lawful, transparent, and fair products that also adhere to the principles such as the one of data minimization and security. Third, we see more and more focus being put on regulating AI (Smuha 2021). In the EU, this has taken the form of the Artificial Intelligence Act (AIA) that the European Commission issued on April 21, 2021, to lay down harmonized rules on AI (see Annex II and Rec. 30). The AIA famously defines AI very broadly, meaning that many of the technologies implemented in SCTs could fall under its scope. It also follows a risk-based approach, meaning that different rules will apply depending on the risks the SCTs pose for individuals (not focused on children specifically).

In the following subsection, we focus on selected topics within those regulatory frameworks to illustrate to what extent the side effects discussed in Sect. 3 are currently being addressed in the EU regulatory framework. We discuss ways beyond these regulations (including suggestions and guidelines provided by academics, NGOs, and parental organizations) that enable taking a more child-centric approach to regulating SCTs.

### Selected deep dives into regulatory frameworks and potential improvements

#### Children’s rights approach: the best interest of the child principle

The goal of the best interest of the child principle is to ensure the full and effective enjoyment of children’s rights. This includes the holistic physical, mental, spiritual, moral, psychological, and social development of children whenever it is likely that actions impact children, including products or services that affect them (United Nations—UN—Committee on the Rights of the Child 2013a, 2013b; Data Protection Commission Ireland 2020; ICO 2020; Children Rights Code 2021). It seems clear from the side effects we have identified that SCTs impact children and may have negative consequences for them.

Note that the best interest principle aims to protect a child's well-being in the broadest sense possible, i.e., not just preventing harms but also empowering children and fostering their development. However, where it is clear that technology is harmful, its application will be against the best interest principle (and presumably against other children's rights). Where harmfulness has not been clearly demonstrated, but there are nevertheless concerns, technology developers should prioritize a precautionary approach (UN Committee on the Rights of the Child 2013a). This implies a better-safe-than-sorry strategy: better not to use technology until it is clear whether it is harmful, how the harms can be mitigated, and how it advances the development of children (Lievens 2010). To implement the best interest principle, companies are required to conduct a child rights impact assessment to concretize the impact on children and to put in place measures that ensure that the impact contributes to children's well-being and that children are certainly not exposed to harm or risks of harm (UN Committee on the Rights of the Child 2013a, b; UNICEF 2021 undated). Such approaches are very much welcomed and should take all the relevant side effects mentioned in Sect. 3 into account. This should hence include a broader outlook on the impact of SCTs and the IoToys more generally especially on the parental/caregiver-child relationship.

UNICEF has developed a child rights impact assessment to guide companies in implementing the best interest principle and other children's rights (UNICEF 2013; see also Mukherjee et al. 2021). The tool accompanies UNICEF's Children's Rights and Business Principles (UNICEF, undated), which among others focus on marketing and advertising. A children's rights approach must focus on individual children, i.e., side effects that impact the individual child and groups of children and children in general (UN Committee on the Rights of the Child 2013a). Hence, the best interest approach is essential in each of the levels addressed in this article. Looking specifically at the side effects we have identified in relation to SCTs, i.e., deception, dependency, social isolation, overtrust, anthropomorphization, and discrimination, it is clear that companies have a responsibility to at least prevent them with respect to children. Clearly, these side effects affect the best interest principle and the right to an optimal development of children (article 6 UNCRC) and—in the latter case—the right to non-discrimination of children (article 2 UN CRC). Moreover, these are not merely children's rights but also three of the four fundamental principles of the UN Convention of the Rights of the Child 1989, the fourth of which is the right of children to be heard (article 12 UNCRC), a right that requires the participation of children in matters that impact them. Given that the right to be heard is inextricably connected to the best interest principle (UN Committee on the Rights of the Child 2013a), it may be argued that children must in some way participate in the development of SCTs. The best interest principle also applies to the topics to be addressed below insofar as there is an impact on children. Either by interpreting established regulation with the best interest principle in mind or by applying norms that pay particular attention to children’s well-being.

#### Data protection

The GDPR provides key rules that apply within the IoToys context. The GDPR recognizes that children deserve a high(er) level of protection within data protection law because they are less aware of the risks and their rights (Recital 38 GDPR).^3^ Accordingly, some provisions of the GDPR specifically mention children and provide additional concerns and obligations for data controllers. However, given the best interests of the child as well as recital 38 of the GDPR, the other provisions of the GDPR should also be interpreted in a way that does justice to the fundamental rights and the wellbeing of children. Moreover, the UN Committee on the Rights of the Child acknowledges that the child right to privacy in Article 16 UNCRC also enshrines a child right to data protection and emphasizes with respect to SCTs that “States parties should ensure that the products and services that contribute to such environments are subject to robust data protection and other privacy regulations and standards” (UN Committee on the Rights of the Child 2021).

##### Transparency

The right to information includes that information must be provided in a concise, transparent, intelligible and easily accessible form, using clear and plain language, in particular when addressing children (Article 12(1) GDPR), which is key for obtaining consent for data processing activities of SCTs. A particular point of attention is when information needs to be communicated to children and their parents; under the GDPR, the first moment is when personal data are obtained from the data subject (Article 13(1) GDPR). However, for SCTs, this is a somewhat challenging provision as it is unknown whether personal data are collected and what happens to it at the time of purchase, although this can be important information when making the purchase decision.

Some work on privacy dashboards for SCT could provide insight into data flows and privacy settings and warn of the consequences of changing those settings. The question then arises whether parents should have access to (all) data flows because that could also infringe on children's privacy vis-à-vis parents, for example, if they have access to all dialogues of their child with a SCT (Keymolen and Van der Hof 2019). Jones and Meurer (2016) suggested that such parental interfaces could display fewer tabs to share their child's conversations on social media. They also propose manufacturers to reveal part of the conversation (that of Barbie's side) without revealing their child's replies. According to them, SCTs should accurately portray their information practices across relationships that are often unclear (i.e., child-to-parents, parents-to-manufacturer, manufacturer-to-third party) through design information sharing (Jones and Meurer 2016).

Still, the strict reliance on an informational perspective of privacy has been criticized, as it neglects the multi-dimensional nature of transparency (Felzmann et al. 2019a, b; 2020). In particular, the performative element of transparency (i.e., how information about data processing is provided) plays an important role. Specifically, companies have used “dark patterns” or misleading designs to obfuscate information retrieval (Gray et al. 2018; Nouwens et al. 2020). This is particularly troubling in the context of SCTs utilized by children, as their awareness of such practices is more limited than adults. Thus, we still have ways to go for the transparency principle of the GDPR to fulfill its ideal in the field of IoToys. Importantly, the transparency principle also has a fairness aspect that should be guiding under the GDPR to ensure that the injustice of an unbalanced power relationship between SCT provider and child/parent is rectified as much as possible (Maglieri 2020). This imbalance is particularly problematic, and potentially unfair, in the case of 'vulnerable data subjects' (Article 29 Working Party—WP29, 2017) under which we should include children, as witnessed by Recital 38 of the GDPR among others.

The data minimization principle could ideally address the challenges of the transparency principle, which is particularly difficult to implement for children (Borenstein et al. 2018). However, there seems to be not much interest from the technical community in achieving data minimization. In this respect, Albuquerque and colleagues (2020) stress that data minimization is rarely mentioned nor addressed in contributions studying SCTs’ privacy requirements. Indeed, some of the literature in this field does not even mention data minimization once (Hung et al. 2016; Jones and Meurer 2016;). In this vein, also the principle of accountability is very much shallowly addressed (Albuquerque et al. 2020).

##### Fairness

As was already mentioned, closely related to transparency is the principle of fairness. At present, the principle of fairness in itself is still somewhat vague, and it should be read in conjunction with other principles, such as lawfulness, transparency, accountability, data minimization, and purpose limitation, as laid down in Article 5 GDPR and elaborated in the subsequent provisions (Clifford and Ausloos 2018; Maglieri 2020). In essence, there must be a fair balance between the interests of the data subject and IoToys companies, whereby the best interests of the child (Article 3 CRC) must be 'a primary consideration' also in the actions of private actors (UN Committee on the Rights of the Child 2013a; b; UN Committee on the Rights of the Child 2021). Taking a child-centered perspective, the principle of fairness also requires broadening the principle, i.e., lowering the threshold of what is considered unfair under the law. One argument for lowering the threshold is that the power imbalances are greater between children and companies than adults and companies (see also WP29 2017 on ‘vulnerable data subjects’). Still, case law or guidance by the European Data Protection Board on how to interpret the principle of fairness in light of children's data processing has yet to emerge (see, however, UN Committee on the Rights of the Child 2021; Data Protection Commission Ireland 2020; ICO 2020; Children Rights Code 2021).

##### Data security

Likewise, the GDPR wants to ensure data processing security, and several provisions throughout the regulation set forth measures to ensure that appropriate steps of IoToys providers are being taken. Art. 32 GDPR mandates IoToys developers to ensure a level of security appropriate to the risk of the processing and lists security mechanisms that need to be implemented. The leading ISO standards on security and the technical literature on security align with security's legal notion (Tamò-Larrieux 2018). How efficiently the principle of data security and the industry standards enforce the security of SCTs cannot be adequately determined. The literature has provided various techniques to address security risks stemming from SCTs themselves (e.g., capturing data emitted by the SCT) as well as security risks enabling access to the collected data (e.g., because of a lack of encrypted communication, breaking into devices, gaining access to passwords) (Rivera et al. 2019; Chaudron et al. 2019). The European Commission also recently issued a “Cybersecurity Strategy for the Digital Decade” (EC 2020b). Part of the strategy is creating an “Internet of Secure Things” environment, which builds upon the Cybersecurity Act (2019), which promotes security solutions and certifications thereof. Envisioned are also “new horizontal rules to improve the cybersecurity of all connected products and associated services placed on the Internal Market. Such rules could include a new duty of care for connected device manufacturers to address software vulnerabilities including the continuation of software and security updates as well as ensuring, at the end of life, deletion of personal and other sensitive data.” (EC 2020b, p. 9). Again, it is important to take into account the specific impact on children and their rights and to develop safety and security safeguards that reflect their situation by performing a children’s rights impact assessment when developing SCTs. Lastly, the Commission recognizes the need for increased education also of children in the field of security and promotes such topics within the Revised Digital Education Action Plan.^4^

##### Right to erasure

An important data subject right under the GDPR is the right of erasure. The underlying rationale of the right to erasure is that a person should be able to start with a clean slate at any time, especially when they are a minor. In other words, people should not spend the rest of their life being confronted with youthful sins (e.g., conversations with SCTs as a child that, later in life, may be seen as embarrassing or as no longer reflecting one's identity). Of course, it depends on how the data are used whether it affects a child. What is relevant, however, is that a person has some degree of control over their personal data and that can be included in the design of SCTs, either by not allowing processing by default or by explicitly giving specific options in a transparent way. In addition, the evolving capacities of children will also have to be taken into account when determining the most appropriate design (Article 5, CRC). With young children it is obvious that some processing should not be allowed by default, and with older children options can be offered to consent to certain data processing within the margins of the GDPR, as long as it is clear to them what the consequences are. Such an option could also be to restrict parents' access to the child's interactions with the toy in order to ensure the privacy of children in that relationship. In any case, children should at some point be able to delete data if it is no longer necessary for the purpose of the processing or consent for processing is withdrawn. Accessible and user-friendly electronic ways of implementing this right can be part of a privacy by design strategy (Van der Hof and Lievens 2018), although realizing this right will prove difficult in AI environments (Fosch Villaronga et al. 2018a, b).

##### Prohibition of automated profiling of children

As was mentioned earlier SCTs can make use of data-driven practices to improve products based on children's data or to draw up consumer profiles of children. Especially with data-driven practices, including profiling, a high level of protection for children will need to be achieved. When automated profiling has a significant effect it is in principle covered by data protection right not to be subject to automated profiling (Article 22 GDPR). The GDPR is not explicit on how the provision should be interpreted with regard to children, although it is likely that a child-centred approach should be adopted (Van der Hof et al. 2020). The GDPR seems to call for a precautionary approach, witnessed by the fact that profiling of children is explicitly mentioned in recitals 38 and 71. It is unfortunate that the high level of protection for children has not been shaped by provisions of the GDPR and particularly Article 22 of the GDPR. WP29 (2017) does, incidentally, indicate that the exceptions to the prohibition of automated decision-making, including profiling, in that provision should be interpreted restrictively with respect to children. This effectively leads to the explanation that children should not be subject to profiling unless it is in the best interest of the child. In any case, the WP29 takes the position that companies should refrain from profiling children for marketing purposes (WP29 2017; see also Van der Hof et al. 2019; see also UN Committee on the Rights of the Child 2021; Data Protection Commission Ireland 2020; ICO 2020; Children Rights Code 2021). Moreover, the Council of Europe goes so far as to say that child profiling should, in principle, be prohibited by law unless it is in their best interests (e.g. contributes to their well-being) or there is an overriding public interest (Council of Europe 2018). When the design of SCTs is driven by commercial interests rather than the best interest of the child, automated profiling should therefore be abandoned. In this case it may be a privacy by design solution to by default turn off any processing of data with the purpose of automated profiling (including the application of its results) in the case of children (Van der Hof and Lievens 2018).

#### Different dimensions of toy safety

The legal definition of safe products is quite broad and it can be understood as covering all kinds of risks that can, directly or indirectly, cause harm to consumers. Traditionally, the definition of safety has been interpreted to apply to risks that have a physical impact on the safety of persons, such as mechanical or chemical risks. However, a growing number of researches support the idea that technology overuse causes ill-being that goes beyond physical safety (Rosen et al. 2014; Carr 2011). In a study including 1030 subjects (338 children, 316 pre-teen, and 376 teenagers), Rosen et al. (2014) concluded that unhealthy eating, lack of physical sport, and technology overuse predicts ill-being. In their study, Rosen et al. (2014) investigated whether certain types of technology, including Internet, email, IMing/chatting, cellphone, video games, music players, or technological toys, had an impact on four different categories of ill-being, including physical problem symptomatology, psychological symptom manifestation, attention problems, and home and classroom behaviors. Children using technology, in particular technological toys, presented total ill-being, attention, and physical problems. On their side, pre-teenagers using email, cell phones, video games, and technological toys also predict more ill-being than those without technology. For teenagers, it appeared that the overuse of any technology significantly predicted ill-being.

Rosen et al. (2014) concluded that technology use might have harmful effects on children and adolescents, and healthier food and more exercise might not be enough to improve their wellbeing. There is no reason to believe why SCTs are going to be excluded from these distracting technologies (Fosch-Villaronga 2019). Policies should adopt an extended concept of safety that encompasses protection against all kinds of risks arising from the product, including cyber-risks (European Commission 2020a, b; Fosch-Villaronga and Mahler 2021). Still, the concept of safety conveys the impression that other aspects such as privacy, data protection, autonomy, psychological harms, diversity, or dignity do not play a role in ensuring a safe human–robot interaction (Holder et al. 2016; Leenes et al. 2017; Fosch-Villaronga 2019). In this respect, more research is needed to grasp whether a safe human–robot interaction can include safeguards to prevent side effects such as social isolation, overtrust, dependency, or deception (Martinetti et al. 2021).

Targeting toys, the Directive on the safety of toys (Directive 2009/48/EC) establishes essential safety requirements (Art. 10). Among others, toys “shall not jeopardise the safety or health of users or third parties when they are used as intended or in a foreseeable way, bearing in mind the behavior of children” (Art. 10(2)). The safety and warning provisions found in the Directive on the safety of toys might have found new momentum as they are explicitly referred to in the proposed AIA. The AIA establishes as ‘high-risk’ those ‘AI systems that pose significant risks to the health and safety or fundamental rights of persons’ (p. 3). According to Rec. 30 AIA, any AI systems that are products that fall within the scope of, for instance, the Directive on the safety of toys will be classified as a high-risk product under the AIA, “if the product in question undergoes the conformity assessment procedure with a third-party conformity assessment body pursuant to that relevant Union harmonisation legislation (listed in Annex II).”

Being qualified as high risk under the AIA comes with many requirements that developers must fulfill, such as the establishment of a risk management system, and ensuring proper data governance. These requirements also refer to the high-quality data, documentation and traceability, transparency, human oversight, accuracy, and robustness, which are strictly necessary to mitigate the risks to fundamental rights and safety posed by AI. However, with respect to SCTs—which is the focus of this article—such a qualification under the high-risk category is unlikely, as safety conformity assessments are only required with respect to “the chemical, physical, mechanical, electrical, flammability, hygiene and radioactivity hazards that the toy may present” (Art. 18 Directive on the safety of toys). This is a huge point of concern given that the contemporary understanding is that these systems need to be safe in physical terms, but also with respect to all the aspects arising from the human-technology interaction (Martinetti et al. 2021).

Nonetheless, the AIA might provide some remedies for the side effects raised by SCTs. But the “remedy” is rather a prohibitive approach: Art. 5 of the AIA prohibits certain products such as SCTs that include AI practices that are likely to cause harm through the manipulation of individuals (including children), the exploitation of vulnerabilities of specific groups of children or persons, or the use of real-time remote biometric identification systems in publicly accessible places. How these restrictions will be altered throughout the legislative process (the AIA is only a proposal at this stage) and how it will be applicable to SCTs remains to be seen.

Research shows individuals suffer from emotional and mental conditions once their personal data has been compromised (Kilovaty 2021). Thus, safety breaches should be defined to go beyond just material or physical or financial breaches. To determine the measures appropriate to the risks, data protection impact assessments (DPIA) can be conducted and are even mandatory for high-risk activities (Article 35 GDPR). On the basis of Recital 38 GDPR, the processing of children's data is a criterion which must be taken into account when determining whether the processing may entail a high risk (Van der Hof and Lievens 2018). This is in line with the WP29 guidelines, which refer to the processing of data relating to vulnerable persons as a criterion (WP29 2017). In addition, innovative use, systematic monitoring and the processing of sensitive data are also relevant criteria which are likely to apply to the IoToys. The UK Information Commissioner’s Office has recommended that a DPIA is indeed carried out in case of the regular or systematic processing of children’s data (ICO 2017). In addition, we recall that the best interest principle (Article 3 CRC) requires that in all actions concerning children their best interests should be a primary consideration. Thus, SCT companies should do a child (rights) impact assessment to investigate the impact of their products on children and their rights and to take action if negative impacts are to be expected (Van der Hof and Lievens 2018). Any such measures must take the evolving capacities of children into account (UN Committee on the Rights on the Child 2013a; b). In practice however, such standardized assessment procedures are lacking, although the ICO’s Age Appropriate Design Code provides an overview of development stages according to the different ages of children (ICO 2020).

#### Commercialization of play

One of the side effects of SCTs identified is that children in a technology-mediated setting almost by definition have to deal with commercial interests. SCTs are simply developed, made and sold by companies that obviously need to earn from them as well. We have already pointed out that in doing so they must also keep the best interests of the child in mind and, indeed, take them into account in the design of their products. In particular, SCTs raise questions in this respect in connection with children's rights to play and leisure in a non-commercial or at least child-friendly environment (Article 31 UNCRC) (see also UN Committee on the Rights of the Child 2013c) and to protection against economic exploitation (Article 32 UNCRC). On top of the considerations already addressed earlier in relation to the best interest of the child and automated profiling of children for commercial purposes, commercialization of play must be a matter of attention because it can, for instance, lead to family conflicts (e.g., because children are pushed to put pressure on their parents to buy products that have been advertised to them or are particularly appealing to them), may be harmful to a child’s development and especially “antithetical to creative play” (at 15) when play is increasingly scripted, or promoting and reinforcing “gender stereotypes or early sexualization of girls” (at 15) (UN Committee on the Rights of the Child 2013a; b).

On top of data protection law providing a high level of protection for children’s personal data in commercial contexts, consumer laws provide rules that aim to prevent or resolve (economic) harms for consumers, in some instances recognizing children as a particularly vulnerable group of consumers. This is the case, for example, in the Unfair Commercial Practices Directive,^5^ where ‘vulnerable consumers’ are awarded an even higher level of protection than the average consumer. Age or credulity being among the factors in determining consumers' vulnerability means that children are included in the concept of 'vulnerable consumer.' Moreover, companies can reasonably expect children to be the users of SCTs since these toys are generally intended for them (foreseeability criterion). In such respect, the literature is rich in examples on how, generally, children tend to anthropomorphize robots (Okanda et al. 2019), assigning human mental states and biological characteristics to robots, even when they have mechanical characteristics (Manzi et al. 2020). Therefore any unfairness of commercial practices will be assessed from the perspective of the average member of the group of children (Article 5(3) Directive 2005/29/EC), which is a particular vulnerable consumer.

Unfair commercial practices generally include practices that mislead or unduly influence consumers, including children. For example, a company may not target a group of consumers, such as children, in a way that exploits specific vulnerabilities that make them easier to deceive than other consumers (ACM 2020). The Directive explicitly prohibits direct exhortations, which entails “putting pressure on children to buy a product directly or to persuade adults to buy items for them (the ‘pester power’)” (European Commission 2016). In addition, there is a more general prohibition of acting contrary to professional diligence (or good entrepreneurial behavior). The company must behave per that professional standard applicable in its business line, which may be grounded in trade practices or codes of conduct. Although the best interest principle in Article 3 CRC is not mentioned as such in Directive 2005/29/EC (it rarely is), the interpretation of the UN Committee of the Rights of the Child is that the principle does apply as a substantive right as well as a principle of interpretation to ensure that the law is interpreted in a child-friendly manner. It also applies as a rule of procedure to ensure, for example, that children have access to justice when their economic interests are harmed or that transparency of commercial practices must be applied in a child-friendly manner (UN Committee on the Rights of the Child 2013a).

In addition to these more traditional forms of commercialization, we see new strategies emerging that can be used to entice children to make purchases, for example, or to make them sensitive to certain brands. With SCTs, commercial messages can be incorporated into the interactions with children for this purpose. What is also new is that the relationship between child and parent on the one hand and the SCT provider on the other has undergone a qualitative change with the networking of toys and the introduction of machine learning into toys never really making the toy one's own (Keymolen and Van der Hof 2019). To this can be added that the children themselves or at least their identity is being commercialized with the for-profit use of their behavioral data and profiles based on it (Verdoodt and Lievens 2017; Van der Hof et al. 2020). The technological turn with respect to toys allows the company to target commercial messages very specifically to children by adapting them to their interests based on the behavioral data of the child, data which can also be sold to third parties (Verdoodt and Lievens 2017). We recall the discussion of profiling in the previous section, showing that, based on the GDPR, clear restrictions, arguably even a prohibition, seem to be placed on profiling children for marketing purposes.

#### Diversity, inclusion, and discrimination

Principle 5 of UNICEF Children’s Rights and Business Principles (undated) puts forward an obligation for business as part of their corporate responsibility to “ensure that products and services are safe, and seek to support children’s rights through them” (at 25). In the European Union, robots may be regulated as products under Directive 2001/95/EC on general product safety and Directive 85/374/EEC on liability for defective products. Product liability rules primarily offer an *ex post* compensation mechanism, but indirectly they also provide incentives for manufacturers to improve, *ex ante,* the safety and security of their products, to avoid liability risks (Expert Group on Liability 2019). The applicability of product liability laws is not straightforward in the context of physically embodied robots comprising cyber-physical systems, i.e. complex and intertangled devices combining “hardware, software, and services” (Noto La Diega and Walden 2016, p. 1).

The UNICEF AI policy guidance formulates several guiding principles, but these principles' formulation remains very vague. For instance, the caption of the requirement 'Prioritize fairness and non-discrimination for children' is not appropriate. The caption reads as follows: "AI must be for all children." Non-discrimination which is one of four fundamental principles under the UN Convention on the Rights of the Child 1989 (Article 2) is not necessarily about AI for all children, but about children not being excluded or treated differently on unfair grounds (van der Hof and Fosch-Villaronga 2020). Moreover, inclusiveness and diversity principles, which indeed are important principles to be taken into account, may also require different adoption and access strategies for children, for example in view of their evolving capacities (Article 5 UNCRC).

With its Principle 2 'Ensure inclusion of and for children', the UNICEF AI policy guidance seems to highlight the importance of inclusive design strategies when stating that "all children should be empowered by AI and play a leading role in designing a responsible digital future for all." However, critical questions of how and to what extent children's inputs are necessary or desirable to 'design a responsible digital future for all' need to be answered (van der Hof and Fosch-Villaronga 2020). Children are not often in a position to understand the magnitude of the problems or risks. Furthermore, parents are sometimes oblivious to those risks, as they are often too emotionally invested in seeing technology as a solution for their child and the technology is usually too complex to understand its use and consequences (Wagner et al. 2018).

#### Societal consequences

Within a best interest view, and beyond the legal remedies discussed earlier, the following suggestions try to tackle these side effects through literacy, institutional, and community initiatives.

The normalization of surveillance can be partly addressed through critical media literacy among parents. Heljakka and Ihamäki (2018) discuss how SCTs require a wide understanding of literacy or a “multi-literacy perspective”, including digital literacy, ludic literacy and transmedia literacy. Concretely, parents should pay attention to the SCT discourse in the media and on social media, reading up about recent developments before purchasing a SCT for their children. Some useful consumer guidelines try to stimulate parents’ reflection when buying a smart toy (Internetmatters 2019; Nash 2018) or for parenting in digital contexts more broadly (Hawkins et al. 2017); in addition, guidelines targeting caregivers and supporting them in choosing SCTs for children in the context of play-based caregiving and development have been proposed (Healey and Mendelsohn 2019). These guidelines advocate for a conscious, engaged and curious parenting style, interested in the lifeworld of the child. Internet Matters (2019) specifically recommends that parents check whether there are lock-in mechanisms with the smart toy such as a monthly subscription. This aspect of commercialization is discussed by Ågren (2020). She found that children in Sweden (aged 4–9) were very aware of the commercial logics of SCTs and games and that the children sometimes used this knowledge to their advantage, for example to persuade their parents to buy them a premium membership in order not to be spammed with ads. Nash (2018) recommends to consult online reviews before buying a SCT, to read the information accompanying the SCT, and to “buy toys from recognised, trusted brands, which might be expected to respond to any observed security flaws”. However, the onus for counteracting the normalization of surveillance should not be squarely on the parents. There is a specific need to “translate” high(er) level policy reports and guidelines on information and media literacy, including privacy as well as the laws pertaining to surveillance, into more concrete and parent-friendly formats.

Beyond parents, schools are a key player and a main institution to tackle some of the societal level side effects. Schools could provide more opportunities for responsible play through the use of suitable SCTs, for example, in project-based learning (Solomon 2003). This could help tackle knowledge gaps between children from different socio-economic backgrounds, addressing digital inequalities and fostering new norms of creativity and play. While most schools have limited freedom to develop curricula, the topic of SCTs—and digital technology more broadly—could be discussed within media literacy, if this is a compulsory school subject, like in Finland (Debating Europe 2020). If this is not the case, SCT awareness could be taught as an example, case or context in other subjects.

Finally, communities, businesses and civil society have a role to play in addressing societal level side effects. Supra-national organizations are particularly suited to draft standards and best practices to address side effects as SCTs are complex products that transcend country and market borders. The International Chamber of Commerce (ICC 2018), for example, as an important business stakeholder, has a specific article in their “Advertising and Marketing Communications” code on children and young people (Article 18). The article addresses aspects such as inexperience and credulity, avoidance of harm, and social values. A specific provision relates to inequalities: “Marketing communication should not suggest that possession or use of the promoted product will give a child or young person physical, psychological or social advantages over other children or young people, or that not possessing the product will have the opposite effect.” Another provision points to the aspects of commodification and commercialization brought up earlier: “Marketing communication should not include any direct appeal to children and young people to persuade their parents or other adults to buy products for them”. Such self-regulation attempts are of course not legally binding but can still encourage best practices for SCT manufacturers and other businesses in the SCT supply chain. Beyond business stakeholders, civil society groups and NGOs in the area of children’s rights could do more to push for a best-interest-of-the-child approach when it comes to SCTs. Such organizations tend to focus their efforts on the protection of children from exploitation, harm and violence, often neglecting other points such as privacy and freedom of expression (UNICEF 2018).

UNICEF's (2018) industry toolkit is an attempt to provide a child rights based set of principles on privacy and freedom of expression. The toolkit includes a checklist with privacy-related questions across four domains (obtaining children’s personal data, using and retaining children’s personal data, ensuring children’s access to information, educating and informing children online). Manufacturers of devices and toys face specific questions such as “Does your company automatically install or push security updates to devices?” and “Does your company incorporate parental control mechanisms in its products, on either an ‘opt-in’ or an ‘opt-out’ basis?” Again, however, this checklist is relatively broad and could do with more specific guidance for manufacturers. Interdisciplinary and multi-stakeholder collaboration could help “translate” between high-level principles and guidelines on the one hand and concrete technical implementation on the other hand.

## Conclusion

In this contribution, we show that the development of toy robots targeting children does not necessarily put their needs at the forefront but that regulatory remedies to address this gap exist. We argue that it is critical to understand in a first step what actual impacts SCTs have on children and the child–parent/caregiver relationship context, to in a second step discuss how current regulations and guidelines can help protect children from unwanted, negative impacts of the IoToys. To do so, we take a socio-legal perspective with the specific lens of protecting children's rights that aims to alert about the potential adverse consequences these systems have for children.

We clustered the side effects into three groups (Fig. 1), loosely following Liu (2018) and acknowledging the interconnectedness of individual level, technical level, and societal level side effects. On the technical level, security vulnerabilities, opacity, and bias were identified as salient side effects. We discussed emotional dependency, manipulation, overtrust, and children's play's commercialization on the individual level. Finally, on the societal level, the normalization of surveillance, additional demands, and burdens on the parents, digital inequalities, and new forms of play and creativity stood out as important side effects. To address the side effects, the overarching principle should be to put children's rights front and center, and this best interest principle permeated our discussion of legal and social remedies to the side effects. We analyzed relevant legislation that partly deals with some side effects (e.g., CRC, GDPR) and studied adjacent legal literature but noticed a lack of specificity and insufficient focus on SCTs. Using a socio-legal lens, we also looked into soft law and non-legal remedies that might alleviate some of the societal side effects. We identified key stakeholders who affect children's use of SCTs (parents, caregivers, communities, businesses, and civil society) and elaborated on strategies that should result in healthier SCT use with fewer side effects.

This article provides an analysis of the arising side effects of SCTs and contrasts them with current regulatory redress mechanisms, highlighting misfits and needs for further policy-making efforts. Taken together, our contribution shows the need to adopt a best interest perspective and to dedicate more attention to SCTs from a socio-legal perspective. Our article hopefully encourages future work to study-specific side effects of SCTs in more depth and develop dedicated and implementable multi-stakeholder strategies to address them.

## Footnotes

1 Barbie has discontinued this project, see https://barbie.mattel.com/shop/en-us/ba/dollhouses.

2 See https://www.fisher-price.com/en_CA/brands/smarttoy.

3 Other (proposed) data protection legislation also includes specific provisions for the protection of children's personal data, see Sect. 33 of the Kenian Data Protection Act 2019 (http://kenyalaw.org/kl/fileadmin/pdfdownloads/Acts/2019/TheDataProtectionAct__No24of2019.pdf) and Sect. 16 of the Draft Personal Data Protection Bill 2019 in India (http://164.100.47.4/BillsTexts/LSBillTexts/Asintroduced/373_2019_LS_Eng.pdf).

4 See https://ec.europa.eu/education/education-in-the-eu/digital-education-action-plan_en.

5 Directive 2005/29/EC of the European Parliament and of the Council of 11 May 2005 concerning unfair business-to-consumer commercial practices in the internal market and amending Council Directive 84/450/EEC, Directives 97/7/EC, 98/27/EC and 2002/65/EC of the European Parliament and of the Council and Regulation (EC) No 2006/2004 of the European Parliament and of the Council (‘Unfair Commercial Practices Directive’) OJ L 149, 11.6.2005, p. 22–39.
